# The evolution of the gut microbiota in the giant and the red pandas

**DOI:** 10.1038/srep10185

**Published:** 2015-05-18

**Authors:** Ying Li, Wei Guo, Shushu Han, Fanli Kong, Chengdong Wang, Desheng Li, Heming Zhang, Mingyao Yang, Huailiang Xu, Bo Zeng, Jiangchao Zhao

**Affiliations:** 1 Farm Animal Genetic Resources Exploration and Innovation Key Laboratory of Sichuan Province, Sichuan Agricultural University, Chengdu, Sichuan 611130, China; 2 China Conservation and Research Center for the Giant Panda, Ya’an, Sichuan 611830 China; 3 College of Animal Science and Technology, Sichuan Agricultural University, Ya’an, Sichuan 625014, China; 4 Department of Pediatrics and Communicable Disease, University of Michigan, Ann Arbor, Michigan 48109, United States of America

## Abstract

The independent dietary shift from carnivore to herbivore with over 90% being bamboo in the giant and the red pandas is of great interests to biologists. Although previous studies have shown convergent evolution of the giant and the red pandas at both morphological and molecular level, the evolution of the gut microbiota in these pandas remains largely unknown. The goal of this study was to determine whether the gut microbiota of the pandas converged due to the same diet, or diverged. We characterized the fecal microbiota from these two species by pyrosequencing the 16S V1–V3 hypervariable regions using the 454 GS FLX Titanium platform. We also included fecal samples from Asian black bears, a species phylogenetically closer to the giant panda, in our analyses. By analyzing the microbiota from these 3 species and those from other carnivores reported previously, we found the gut microbiotas of the giant pandas are distinct from those of the red pandas and clustered closer to those of the black bears. Our data suggests the divergent evolution of the gut microbiota in the pandas.

The advent of the high throughput next generation sequencing has allowed scientists to explore the gut microbiota with an unprecedented depth. The evolution of the gut microbiota has recently received great interests^1^,^2^,^3^. Several factors such as diet and phylogeny have been reported to play important roles in shaping the gut microbiota at different taxonomic scales^2^,^3^,^4^,^5^,^6^,^7^.

The red and the giant pandas are interesting models to study the evolution of the gut microbiota as they are carnivores by phylogeny but herbivores by diet. Both species experienced a dietary switch from carnivores to highly specialized bamboo eaters. They both independently developed several similar morphological features such as the false thumb^8^ in adaptation to the same dietary switch to bamboo. However, it is still unclear whether the gut microbiota in the red and the giant pandas converged due to the similar, highly specialized bamboo diet, or diverged corresponding to other unknown factors.

To determine their evolutionary patterns we characterized the gut microbiotas in the two types of pandas and compared them to the gut microbiotas of black bears. We found a divergent evolution pattern in the gut microbiotas of the pandas.

## Results

### Comparison of the gut microbiota of the three carnivores

We characterized the gut microbiotas of 6 red and 5 giant pandas by sequencing the 16S V1–V3 hyper variable region of their feces collected from the zoo. We also sequenced the gut microbiotas of 6 Asian black bears, which are phylogenetically closer to the giant panda than to the red panda. The sequences were processed and analyzed by using the mothur software package^9^. We retained a total of 63, 944 high quality reads after denoising, with an average of 3,761 sequences per sample ranging from 1,214 to 7,450. These sequences were assigned to 235 operational taxonomic units (OTUs). Sequence number for each sample was normalized to 1,200 by randomly subsampling to minimize the biases generated by sequencing depth. The average ± SD Good’s coverage was 99.3 ± 0.6% (Table S1).

**Figure 1:**
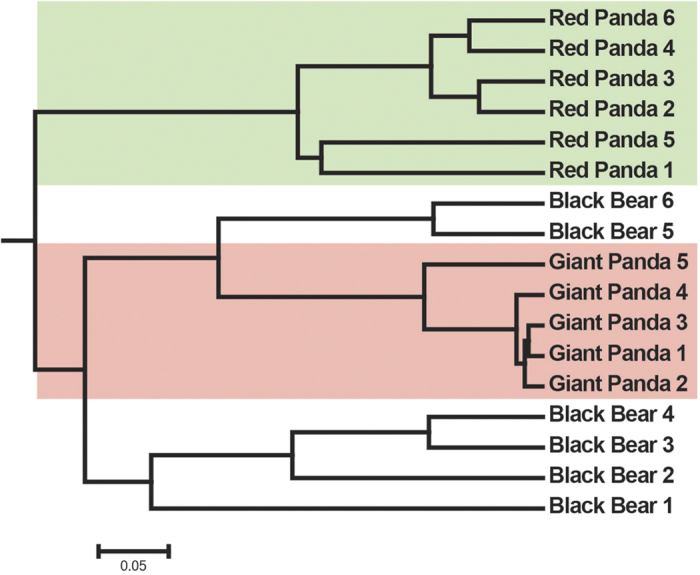
Clustering analysis of the evolution of the gut microbiotas of the black bears, the giant and the red pandas. Gut microbiota trees were generated using the Unweighted Pair Group Method with Arithmetic Mean algorithm based on the Bray-Curtis distances generated by mothur.

We examined the relationship between the gut microbiotas from different species by using Bray-Curtis distances^10^, which were visualized by a dendrogram. Each branch on the tree represents one gut microbiota (Fig. 1). Interestingly, the gut microbiotas of the giant pandas located on different branches from those of the red pandas and clustered closer to those of the black bears (Fig. 1).

**Figure 2:**
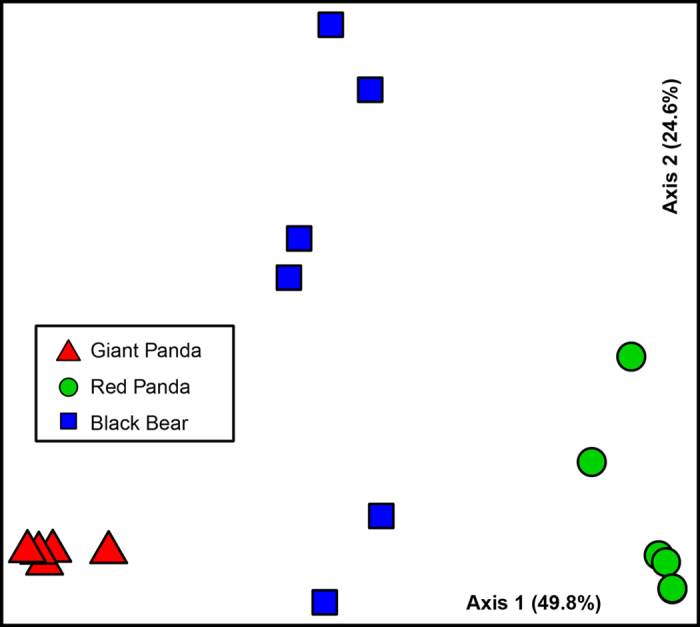
Principal coordinate analysis of the community structure using Bray-Curtis distances. Green circles, blue squares and red triangles represent the gut microbiotas from the red pandas, the black bears and the giant pandas, respectively. Distances between symbols on the ordination plot reflect relative dissimilarities in community structures.

We also used principal coordinate analyses (PCoA) to examine the relationships between gut microbiota of the red and the giant pandas (Fig. 2). We observed similar clustering patterns. On the PCoA plot, each symbol represents one gut microbiota. Consistent with the dendrograms, the gut microbiotas of the giant pandas were distinct from those of the red pandas. Analysis of molecular variance (AMOVA) of the Bray-Curtis distances proved that the differences between the gut microbiotas of the giant and the red pandas were statistically significant (AMOVA, P < 0.05). Interestingly, the gut microbiota of the giant pandas clustered closer to the gut microbiota of the black bears than to the red pandas, consistent with the phylogenetic relationships between the pandas and the black bears (Fig. 2). Analysis of similarity (ANOSIM) supported the PCoA result. The r value (ANOSIM, r = 0.93, P < 0.05) between the giant pandas and the red pandas is larger than that (ANOSIM, r = 0.59, P < 0.05) between the giant pandas and the black bears (Table S2).

These observations were also supported by other measures of distance metrics such as the ThetaYC^11^, the weighted Unifrac^12^ and the Morisita-Horn^13^ (Figure S1 and S2).

### Unique and shared bacterial taxa in the giant and the red pandas

**Figure 3:**
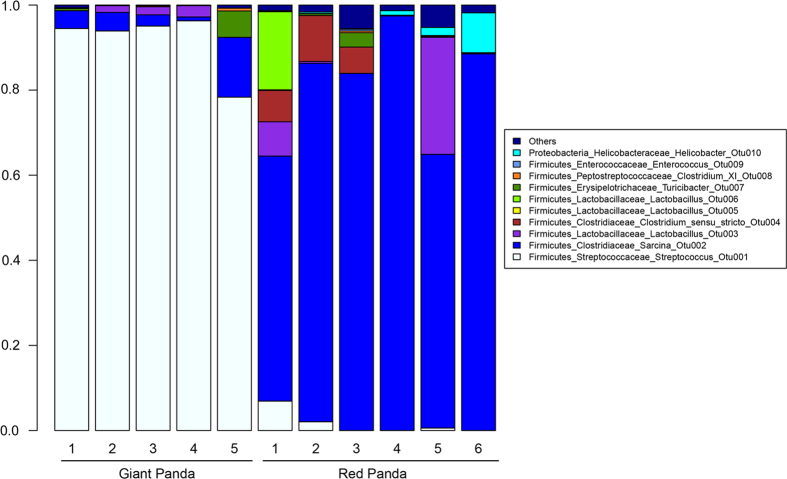
Relative abundance of OTUs at the genus level in the gut microbiotas from the giant and the red pandas. Each bar in the stacked bar charts represents the relative abundance of an individual OTU.

**Figure 4:**
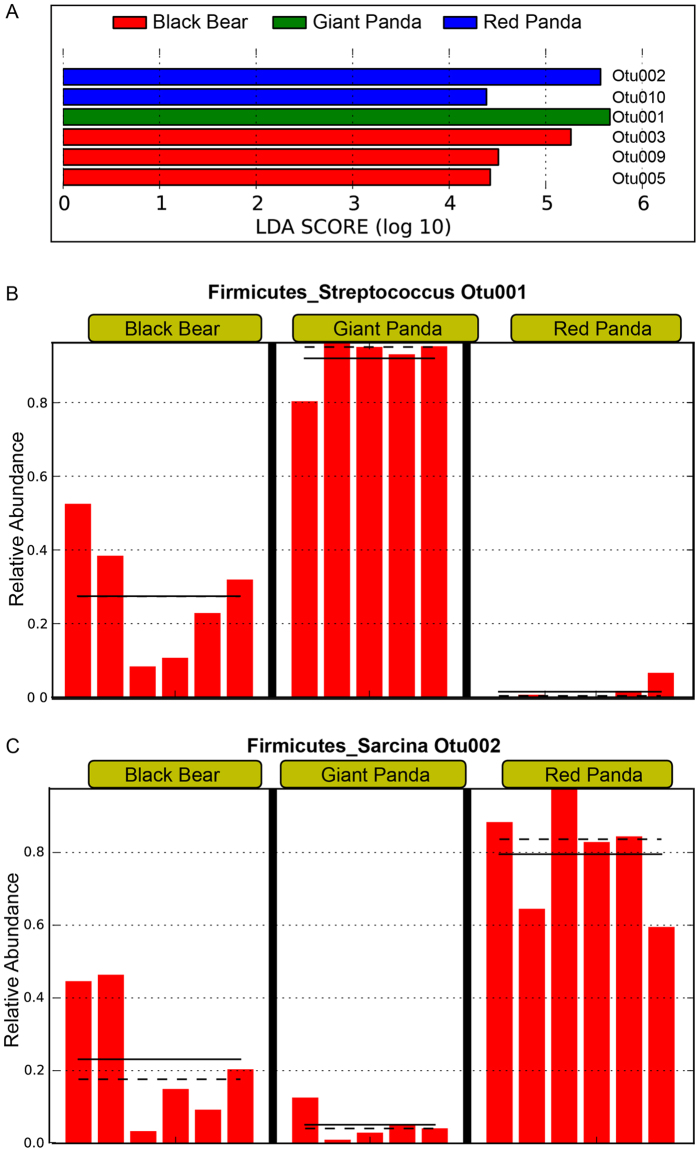
OTUs differentially represented between the black bears, the giant and the red pandas identified by linear discriminant analysis coupled with effect size (LEfSe). **A**. Histogram showing operational taxonomic units (OTUs) that are more abundant in the red pandas (blue color), the black bears (red color) or the giant pandas (green color) ranked by linear discriminant analysis (LDA) score. The relative abundance of OTU001 (more abundant in the giant pandas) and OTU002 (more abundant in red pandas) are illustrated in **B** and **C**, respectively. The mean and median relative abundance of these OTUs are indicated with solid and dashed lines, respectively.

We next sought to examine the shared and unique bacterial taxa between the gut microbiotas of the giant and the red pandas using our sequencing data. The distributions of the top 10 OTUs of the giant and the red pandas are shown in Fig. 3. We used linear discriminant analysis effect size (LEfSe)^14^ to identify OTUs differentially represented between the red and the giant pandas. While OTU001 and OTU002 were shared by all the pandas, their relative abundances were differentially represented between the two species of pandas (Fig. 4A). OTU001 is affiliated with the genus *Streptococcus* and significantly higher in the giant pandas than in the red pandas (Fig. 4B). In contrast, OTU002, classified as *Sarcina*, a member of the *Clostridiaceae* family, is more abundant in the red pandas (Fig. 4C). Both OTUs were also observed in black bears, but with intermediate relative abundance between the two pandas (Fig. 4B,C). OTU003 was found in 3/5 of the giant pandas and 4/6 of the red pandas and belonged to the genus of *Lactobacillus*. OTU10 (*Helicobacter*) was only observed in the red pandas and was absent from the giant pandas. Whether or not the gut microbiotas are involved in the digestion of the highly fibrous diet needs further investigation.

## Discussion

The evolution of the mammal gut microbiotas are affected by several factors. Muegge *et al*^3^, for example, showed that diet has played important roles in the evolution of the gut microbiota, i.e. the gut microbiotas of the carnivores are distinct from those of the herbivores and omnivores. Since both the red pandas and the giant pandas have evolved to adapt to the same, highly specialized diet (bamboo), it is easy to postulate that they share similar gut microbiota. However, our study suggests that despite sharing the same diet, the red pandas and the giant pandas harbor different gut microbiotas. Both the dendrogram and PCoA plot support the divergent evolution of the gut microbiota of these two pandas.

Phylogeny is another factor driving the evolution of the structure of gut microbiotas as reported by several recent studies^2^,^4^,^15^. To put our study into a phylogenetic context, we incorporated our data with the gut microbiotas of several other carnivores reported by Muegge *et al.*^3^, although not ideal due to different DNA extraction methods and regions of the 16S rDNA gene. Nevertheless, we observed similar divergent patterns of the gut microbiotas in the giant and the red pandas in the combined data set (Figure S3). In a recent study, we reported distinct gut microbiotas in the wild and the captive red pandas^16^. Interestingly, after incorporation of the wild red panda gut microbiota into this study, significant differences in the gut microbiotas between the giant panda and the wild red pandas were also observed (Figure S4, AMOVA, P < 0.001). Of note, although the divergent evolution of the gut microbiotas in pandas is consistent with their host’s phylogeny, further experiments including more species are required to test this hypothesis. Other unknown host and environmental factors may have also contributed to the divergent evolution of the gut microbiotas in these pandas.

Contrary to a recent study^17^, which showed diverse bacterial communities belonging mainly to Gammaproteobacteria in four giant pandas, we identified gut microbiotas with much lower diversity and dominated mainly by Firmicutes in the giant and the red pandas. Inconsistency in the gut microbiotas of pandas was also observed in another study^1^, which examined the gut microbiotas in mammals by sequencing the clone libraries of the 16S gene and showed that the gut microbiota of the giant panda was dominated by Firmicutes while the gut microbiotas of the two red pandas consisted mainly of Gammaproteobacteria. The discrepancies between these studies might be attributed to the differences in DNA extraction, hypervariable regions of the 16S rDNA gene, sequencing method and depth, environment and/or other host physiological and genetic factors. Of note, the community diversities of these pandas are low, dominated by one or two OTUs, likely due to their highly specialized fibrous diet (bamboo) with antibacterial activities^18^.

One striking feature of the pandas is their unique bamboo-specialized diet. However, both the giant and the red pandas have short and relatively simple digestive tract and cannot process bamboo efficiently by themselves^19^, especially the cellulose of the cell walls. Recent culture-independent studies have suggested the presence of cellulose degraders in both giant^20^ and red pandas^16^. It is very possible that although the giant and the red pandas possess overall different gut microbiota, they do share certain cellulose degraders or degradation pathways that have converged to help with their digestion of bamboo. Future studies using culture based (e.g. cellulose media) or metagenomics (i.e. sequencing the collected gut microbiota instead of just the 16S rRNA gene) based approaches are desired to address this question.

In summary, we characterized the gut microbiotas of the red and the giant pandas and found that, according the 16S rRNA based community structure analysis, the gut microbiota of these pandas diverged rather than converged based on the same diet. We also identified bacterial taxa deferentially represented between the two species of pandas. More studies are desired to examine their roles in the hosts’ physiology, development, health and disease.

## Methods

### Ethics Statement

Fecal samples were collected from captive and wild red pandas and black bears in Bifengxia Ecological Zoo (Ya’an, Sichuan Province, China). Fecal samples of the giant pandas were collected in China Conservation and Research Center for the Giant Panda, Ya’an, Sichuan Province, China. All the samples were collected by experienced trackers and were immediately frozen in a liquid nitrogen container before transferred to and stored at −80 °C. All animal work was approved by the Institutional Animal Care and Use Committee of the Sichuan Agricultural University under permit number DKY-B20130302.

All experiments were performed in accordance with the approved guidelines and regulations.

### DNA extraction and pyrosequencing

Frozen fecal samples were thawed on ice and dissected. To avoid soil contamination, DNA was then extracted from the inner part of the fecal samples (0.25 g) using the MO BIO PowerFecal™ DNA Isolation Kit (MO BIO Laboratories, Carlsbad, CA, USA) according to the manufacturer’s instructions and the DNA concentration was measured by using Nanodrop (Thermo Scientific). DNA pyrosequencing was performed at the Beijing Genomics Institute (BGI Shenzhen, China) via 454 Life Sciences/Roche GS FLX Titanium platform. Briefly, DNA was amplified by using the V1–V3 hypervariable regions of the bacterial 16S rRNA gene bar-coded primers (forward: CCGTCAATTCMTTTGAGTTT, reverse: ACTCCTACGGGAGGCAGCAG). The PCR reaction (50 μl) contained 50 ng DNA, 41 μl molecular biology grade water, 5 μl 10 x FastStart High Fidelity Reaction Buffer containing 18 mM MgCl_2_, 1 μl dNTPs (10 mM each), 1 μl Fusion Primer A (10 mM), 1 μl Fusion Primer B (10 mM), and 1 μl FastStart High Fidelity Enzyme Blend (5 U/ml). PCR cycles included 95 ^o^C for 2 min; 30 cycles of 95 ^o^C for 20 s, 50 ^o^C for 30 s, and 72 ^o^C for 5 min; and a final extension at 72 ^o^C for 10 min.

### Sequence analysis

Sequencing reads were processed and analyzed using mothur v1.34 following the 454 SOP^21^ on the mothur wiki webpage ( http://www.mothur.org/wiki/454_SOP) on January 7, 2015. After several steps of denoising by using the PyroNoise^22^, Uchime^23^, and preclustering methods^24^, high quality sequences that had a length of at least 200 bp and without sequencing errors or chimeras were retained and assigned to OTUs using an average neighbor algorithm with a 97% similarity cutoff. OTUs were classified at the genus level using the Bayesian method^25^. The number of reads per sample was randomly subsampled to 1,200 to minimize biases caused by sequencing depth. Subsampling to the smallest number of reads was also performed for the two data sets incorporating the wild red pandas and the carnivores from Muegge *et al.*, respectively. For the later, sequences were trimmed to overlap with their data during the alignment step.

Good’s coverage and beta diversity measures including Bray-Curtis, Morisita-Horn, Weighted Unifrac and ThetaYC distances were calculated using mothur. These beta diversity metrics were used to assess the dissimilarity between the communities’ structures. Gut microbiota trees were generated using the Unweighted Pair Group Method with Arithmetic Mean algorithm based on the different distance metrics generated by mothur.

### Statistical methods

Linear discriminant analysis effect size (LEfSe), which takes into account both statistical significance and biological relevance, was conducted to identify OTUs differentially represented between the red and the giant pandas. A P < 0.05 was considered statistically significant.

### Accession numbers

The raw sequences of this study have been deposited in the Sequence Read Archive (accession number SRR1766294). Part of the sequencing data has been published elsewhere to compare the gut microbiota in the wild and the captive red pandas^16^.

## Author Contributions

Conceived and designed the experiments: Y.L. and J.Z., Performed the experiments: Y.L., J.Z. and W.G., Contributed reagents/materials/analysis tools: S.H., F.K., C.W., D.L., H.Z., B.Z., H.X., and M.Y., Wrote the paper: J.Z. and Y.L.

## Additional Information

**How to cite this article**: Li, Y. *et al*. The evolution of the gut microbiota in the giant and the red pandas. *Sci. Rep.* **5**, 10185; doi: 10.1038/srep10185 (2015).

## Supplementary Material

Supplementary Information
