# Supplementary material for: The evolution of the gut microbiota in the giant and the red pandas
Source: Sci Rep. 2015 May 18;5:10185. doi: 10.1038/srep10185 (PMC4434948; doi:10.1038/srep10185)
Supplement: Supplementary Information [file srep10185-s1.pdf]

The evolution of the gut microbiota in the giant and the red pandas

Ying Li[1], Wei Guo[1], Shushu Han[1], Fanli Kong[1], Chengdong Wang[2], Desheng Li[2], Heming Zhang[2], Mingyao Yang[1], Huailiang Xu[3], Bo Zeng[1] and Jiangchao Zhao[4]

Supplementary material

Figure S1

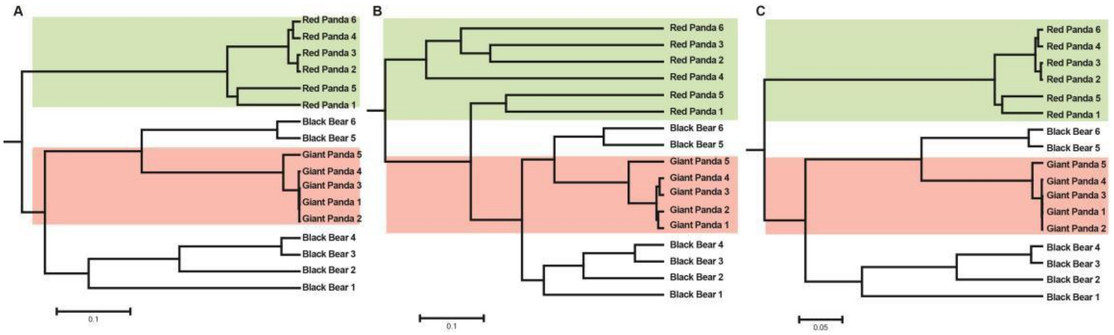

Figure S1. Clustering analysis of the evolution of the gut microbiotas of the black bears, the giant and the red pandas. Gut microbiota trees were generated using the Unweighted Pair Group Method with Arithmetic Mean algorithm based on the Theta YC (A), Weighted Unifrac (B) and Morisita-Horn (C) distances generated by mothur.

Figure S2

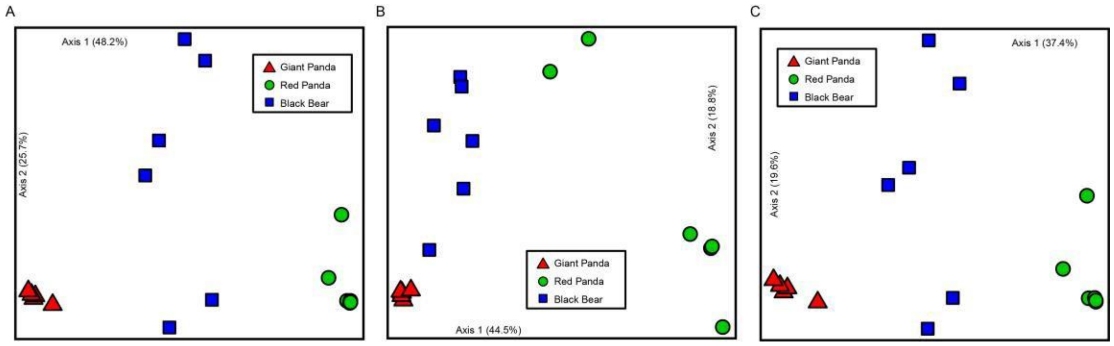

Figure S2. Principal coordinate analysis of the community structure using the ThetaYC (A), Weighted Unifrac (B) and Morisita-Horn (C) distances. Green circles, blue squares and red triangles represent the gut microbiotas from the red pandas, the black bears and the giant pandas, respectively. Distances between symbols on the ordination plot reflect relative dissimilarities in community structures.

Figure S3

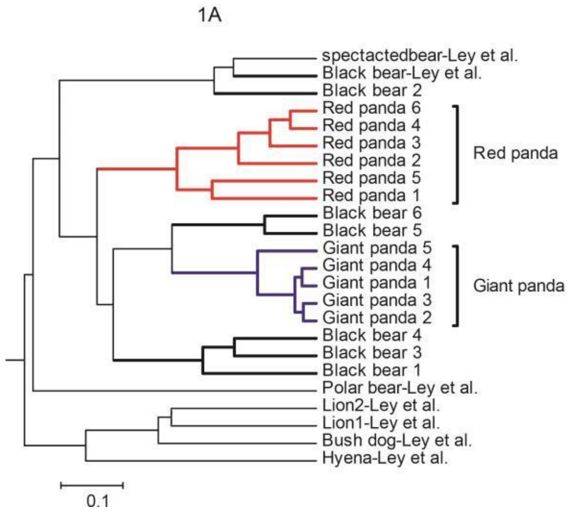

Figure S3. Clustering analysis of the evolution of the gut microbiota (A) and their hosts (B). Gut microbiota trees were generated using the Unweighted Pair Group Method with Arithmetic Mean algorithm based on the Bray-Curtis distances generated by mothur. The host tree was drawn manually based on the report of Flynn *et al*[1] (2005).

Figure S4

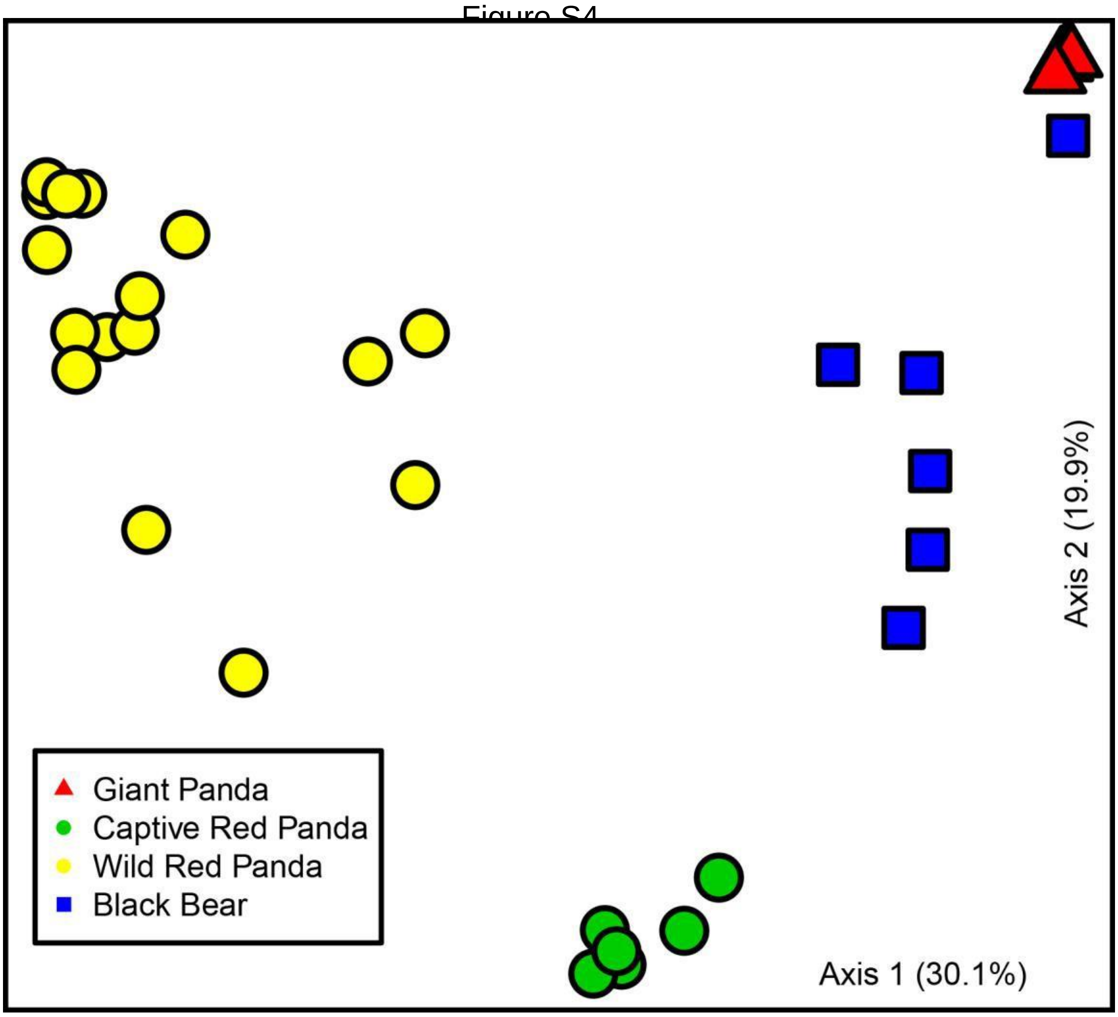

Figure S4. Principal coordinate analysis of the community structures using Bray-Curtis distances. Green circles, yellow circles, blue squares and red triangles represent the gut microbiotas from the captive red pandas, the wild red pandas, the black bears and the giant pandas, respectively. Distances between symbols on the ordination plot reflect relative dissimilarities in community structures. Other symbols represent the gut microbiotas from other carnivores reported elsewhere.

Table S1 Number of sequences and Good’s coverage et al.

Label	group	nseqs	Good's Coverage	Shannon Diversity	# OTUs
17	Captive Giant Panda1	5452	0.999167	0.227366	6
18	Captive Giant Panda2	4831	0.9975	0.321613	7
19	Captive Giant Panda3	5019	0.998333	0.258643	7
20	Captive Giant Panda4	5467	0.998333	0.197337	5
21	Captive Giant Panda5	4139	0.995833	0.706565	10
56	Black Bear1	5601	0.9975	1.329881	10
57	Black Bear2	2443	0.9825	2.280979	40
58	Black Bear3	5935	0.996667	1.045388	11
61	Black Bear4	7450	0.995	0.888016	16
63	Black Bear5	2445	0.9925	1.280523	18
64	Black Bear6	3493	0.998333	0.833749	6
66	Captive Red Panda1	2873	0.9975	1.289361	11
67	Captive Red Panda2	1563	0.990833	0.669335	20
68	Captive Red Panda3	1389	0.976667	0.998137	47
69	Captive Red Panda4	1214	0.986667	0.244044	19
70	Captive Red Panda5	3065	0.989167	1.033259	22
71	Captive Red Panda6	1565	0.989167	0.52532	19

Table S2 Analysis of Similarity of the Bray-Curtis distances

	Black Bear	Giant Panda	Red Panda
Black Bear	0	0.59	0.71
Giant Panda	0.59	0	0.93
Red Panda	0.71	0.93	0

1. Flynn, J. J., Finarelli, J. A., Zehr, S., Hsu, J. & Nedbal, M. A. Molecular phylogeny of the carnivora (mammalia): assessing the impact of increased sampling on resolving enigmatic relationships. *Systematic biology* **54,** 317–337 (2005).
